# Accuracy of the Multisensory Wristwatch Polar Vantage's Estimation of Energy Expenditure in Various Activities: Instrument Validation Study

**DOI:** 10.2196/14534

**Published:** 2019-10-02

**Authors:** Rahel Gilgen-Ammann, Theresa Schweizer, Thomas Wyss

**Affiliations:** 1 Swiss Federal Institute of Sport Magglingen Magglingen Switzerland

**Keywords:** validation, mHealth and eHealth, activity monitor

## Abstract

**Background:**

Sport watches and fitness trackers provide a feasible way of obtaining energy expenditure (EE) estimations in daily life as well as during exercise. However, today’s popular wrist-worn technologies show only poor-to-moderate EE accuracy. Recently, the invention of optical heart rate measurement and the further development of accelerometers in wrist units have opened up the possibility of measuring EE.

**Objective:**

This study aimed to validate the new multisensory wristwatch Polar Vantage and its EE estimation in healthy individuals during low-to-high-intensity activities against indirect calorimetry.

**Methods:**

Overall, 30 volunteers (15 females; mean age 29.5 [SD 5.1] years; mean height 1.7 [SD 0.8] m; mean weight 67.5 [SD 8.7] kg; mean maximal oxygen uptake 53.4 [SD 6.8] mL/min·kg) performed 7 activities—ranging in intensity from sitting to playing floorball—in a semistructured indoor environment for 10 min each, with 2-min breaks in between. These activities were performed while wearing the Polar Vantage M wristwatch and the MetaMax 3B spirometer.

**Results:**

After EE estimation, a mean (SD) of 69.1 (42.7) kcal and 71.4 (37.8) kcal per 10-min activity were reported for the MetaMax 3B and the Polar Vantage, respectively, with a strong correlation of *r*=0.892 (*P*<.001). The systematic bias was 2.3 kcal (3.3%), with 37.8 kcal limits of agreement. The lowest mean absolute percentage errors were reported during the sitting and reading activities (9.1%), and the highest error rates during household chores (31.4%). On average, 59.5% of the mean EE values obtained by the Polar Vantage were within ±20% of accuracy when compared with the MetaMax 3B. The activity intensity quantified by perceived exertion (odds ratio [OR] 2.028; *P*<.001) and wrist circumference (OR −1.533; *P*=.03) predicted 29% of the error rates within the Polar Vantage.

**Conclusions:**

The Polar Vantage has a statistically moderate-to-good accuracy in EE estimation that is activity dependent. During sitting and reading activities, the EE estimation is very good, whereas during nonsteady activities that require wrist and arm movement, the EE accuracy is only moderate. However, compared with other available wrist-worn EE monitors, the Polar Vantage can be recommended, as it performs among the best.

## Introduction

### Previous Research

The accurate measurement of human body energy expenditure (EE) is an important parameter for many applications [1,2]. For example, the intensity of physical activity can be evaluated based on the energy consumed during exercise, and dietary guidance can be given based on the total daily EE [3].

The exact measurement of human EE requires laboratory methods that are not suitable for performing in everyday life, such as wearing a face mask that measures respiration gases or analyzing saliva or urine samples using expensive doubly labeled water techniques [4]. Body-worn sensors provide a consumer-friendly option for measuring EE in daily life and during exercise; originally, these sensors used heart rate (HR) to estimate EE during exercise [5,6] or were assisted by accelerometer-based measurements. Recently, however, the invention of optical HR measurement and the further development of accelerometers in wrist units have opened up the new possibility of feasibly measuring EE in exercise and in daily life [4,7]. These technologies rely on photoplethysmography and use HR-derived algorithms to contribute to the estimation of EE based on activity intensity [8,9]. However, previous validation studies on a variety of body-worn sensor brands reported error rates of approximately 10% to 210%, with more accurate values measured during high-intensity aerobic exercises, such as running or cycling, than during daily activities of low-to-moderate intensity, such as lying down or sitting [10-13]. The limitations of such sensors include distorted optical pulse signals because of motion artifacts, the inability of the devices to account for additional load carried by the user, and distal sensor placement on the body [7,14].

### This Study

Smart Calories is a novel EE calculation by Polar that aims to improve the EE estimation in daily life and exercise. The Polar Vantage (V and M) is a multisensory wrist-worn technology that expresses EE in calories per activity. This new measurement system has not been validated before. Therefore, the aim of this study was to validate Polar Vantage and its EE estimation in a healthy and heterogeneous sample at rest and during different exercise modes and at different intensities against the criterion of indirect calorimetry measure.

## Methods

### Participants

A total of 30 healthy and lean volunteers gave informed consent to participate in this study. None of the participants were known to be taking any medications affecting HR or metabolism nor did they have any tattoos on the nondominant wrist. Of the participants, 50% (15/30) were female and all were within the age range of 20 to 40 years. In terms of activity levels, 33% (10/30) of the participants met or nearly met the physical activity guidelines (ie, completing 150 min of moderate-intensity activity per week), 33% (10/30) of the participants were active (ie, participating in regular training but with no competitive targets), and 33% (10/30) of the participants were endurance athletes (ie, regularly participating in running, triathlon, or cycling competitions). The participants completed written informed consent forms and physical activity readiness questionnaires (PAR-Q) before taking part in the study. None answered *yes* to any PAR-Q question. For sample size estimation, the data of a similar study and similar expected monitor accuracy were used as the estimate for a paired 2-tailed *t* test [13]. Approval for this study was granted by the ethics commission of the Canton Berne (2018-00309), and it conformed to the principles of the Declaration of Helsinki.

### Procedure

The measurements were taken on 2 separate test days. On day 1, the resting HR, maximal oxygen uptake (VO_2max_), and maximal heart rate (HR_max_) were obtained in a laboratory. The participants were scheduled during the morning hours, and they were instructed to avoid any strenuous physical activity and caffeine intake for a minimum of 24 and 12 hours, respectively, before the appointment. First, information about the study was verbally repeated to the participants, and both the questionnaires and informed consent form were confirmed. Second, the body weight (of the participant in underwear), body height, wrist circumference, skin color (using the Fitzpatrick scale from 1 lightest tone to 6 darkest) [15], and skin hair on the wrist (0=little hair, 1=moderate or a lot of hair) were assessed by the supervisor. Third, an HR strap was mounted around the participant’s chest. Then, the HR was measured for 10 min with the participant in a supine position and completely at rest in a quiet and thermoneutral environment (20°-22° C) [16]. Thereafter, the VO_2max_ and HR_max_ assessments were conducted on a treadmill (h/p/cosmos pulsar; Cosmos Sports & Medical Ltd). Initially, a warm-up and treadmill familiarization period of 5 min at 8.6 km/h and 0% inclination was conducted, followed by a short rest period during which a spirometer face mask was fixed on the participant. To determine the VO_2max_ and HR_max_, a graded protocol from an initial speed of 7.5 km/h and a 7% constant inclination was applied, with a speed increase of 0.5 km/h every 30 seconds until voluntary exhaustion [17]. Immediately after voluntary exhaustion, the participant was asked to rate the perceived exertion using the Borg scale (6-20) [18]. For the determination of VO_2max_, at least 2 of the following 4 maximum criteria had to be fulfilled: respiratory exchange ratio greater than 1.1, voluntary maximum (Borg scale *≥* 18), plateau in VO_2_, or HR greater than 85% HR_max_ (HR_max_ estimation based on 220 bpm minus the participant’s age in years) [17].

On day 2, the measurements of EE values during rest, daily activities, and sport activities were obtained in a gym hall with prepared areas. The participants were scheduled anytime during the day, 2 hours after the last food intake, and 12 hours after the last caffeine intake. The participants were equipped with the Polar Vantage M, the Polar H10 chest strap, the MetaMax 3B data logger, and a spirometer face mask. Following a short recreational walk of 3 min as a warm-up, the participants performed 7 simulated free-living activities for 10 min each. After each activity, the participant walked to the next activity and stood still for the remainder of the 2-min break; however, following the running activity, the break lasted for 4 min. During these recovery phases, the upcoming activity was explained. The starting and stopping time of each activity task was registered by the researcher on a paper version of the study protocol, using a master stopwatch. Moreover, when each activity was started and stopped, the respective EE values given by the Polar Vantage M for each activity were recorded.

The 7 activities and their order were as follows:

1. Sitting in a chair and reading (sedentary activity; training mode *other indoor*).
2. Wiping the floor with a mop and hanging out the laundry at a self-guided order and pace (household chores; training mode *other indoor*).
3. Normal walking on an indoor round track of 290 meters with the pacing instruction “as you would go to the bus station in no rush” (gait activity; training mode *walking*).
4. Jogging on an indoor round track of 290 meters with the pacing instruction “choose your own pace at which you could talk to someone” (gait activity; training mode *running*).
5. A strength training circuit of 45-second workouts with a dumbbell in each hand followed by 15-second rests, including squats, shoulder shrugs, bicep curls, lunges, and sit-ups [19] with the instruction “choose your own dumbbell weight and pacing so that the workout is at least somewhat hard” (Borg scale value >12; sport activity; training mode *strength training*).
6. Cycling on an ergometer (Ergoselect 200; Ergoline GmbH) at 80 rounds per min and an HR around 120 beats per min (sport activity; training mode *indoor cycling*).
7. A floorball course (approximately 80 meters in length) including drippling, passing the ball, shooting, and jogging [20], for which the task execution was self-paced but short recovery phases of 10 seconds slow walking per round were required (sport activity; training mode *other indoor*).

Immediately after the termination of each task, individual Borg scale values were reported to rate the perceived exertion [18].

### Instruments

The investigated device was the Polar Vantage M wristwatch (Polar Electro Oy), which uses a bioimpedance-assisted optical HR calculation and 3D acceleration signal. The Polar Vantage M was placed on the participant’s nondominant wrist, 1 finger behind the wrist bone. The participant’s anthropometrics, resting HR, HR_max_, and VO_2max_ values were entered into the user profile, and each corresponding training mode was set in the user setting before starting the respective activity. HR was assessed using the Polar H10 chest strap [21]. To obtain measures of oxygen consumption (VO_2_) and carbon dioxide production (VCO_2_) to determine the VO_2max_, the Quark CPET (Cosmed) was used. To calculate the EE criterion during simulated free-living activities, the VO_2_ and VCO_2_ were obtained using a portable open-circuit metabolic system (MetaMax 3B; Cortex Biophysik) [22,23]. The MetaMax 3B was mounted on the participant with a face mask and a chest harness. All devices were calibrated before each measurement according to the manufacturer’s instructions.

### Data Processing

Each participant’s resting HR was calculated based on the average minimum 30-second values obtained during the 10-min resting measurement, whereas the VO_2max_ and HR_max_ were calculated based on the average maximal 30-second values obtained during the graded treadmill test [17].

To investigate the EE estimations on measurement day 2, the EE values shown on the Polar Vantage M display were noted for each single 10-min activity. To calculate the EE criterion, the formula presented by Elia and Livesey [24] was used to sum up the gas exchange data in kilocalories per minute to generate the total EE per 10-min activity [25]. Each of the 30 participants completed all 7 activity tasks, but 2 technical failures during the floorball course were reported. Therefore, of the 210 activities, 208 were recorded and analyzed (99%).

### Statistical Analysis

Descriptive statistics with mean absolute and percentage errors, Pearson correlations, Bland and Altman analysis, and EE 20% accuracy were used. Bland and Altman analyses with corresponding 95% limits of agreement (SD 1.96) were used to calculate and visualize systematic differences in the EE estimations [26]. The EE 20% accuracy was defined as the percentage at which the Polar Vantage M was within the proposed equivalence zone of ± 20% from the criterion values [12,19,27]. Moreover, backward multiple linear regression analyses with the mean absolute error (MAE) as the dependent variable were performed to investigate potential confounding effects of the independent variables of gender, body mass index, wrist circumference, skin color, wrist hair, HR, resting HR, HR_max_, VO_2max_, and perceived exertion (Borg scale value) on EE accuracy. In addition, backward multiple linear regression analyses were performed on clustered activity groups: (1) low-to-moderate-intensity activities (sitting and reading, household chores, and walking) and high-intensity activities (jogging, strength training circuit, cycling, and floorball course) and (2) activities with no-to-little (steady) arm movement (sitting and reading, walking, jogging, and cycling) and activities with a lot (unsteady) of arm movement (household chores, strength training circuit, and floorball course). In the case of multicollinearity (*r* ≥0.80) or the nonsignificant prediction of the MAE, the relevant variable was excluded from the regression analysis. Any *P* value less than .05 was considered statistically significant.

## Results

**Figure 1:**
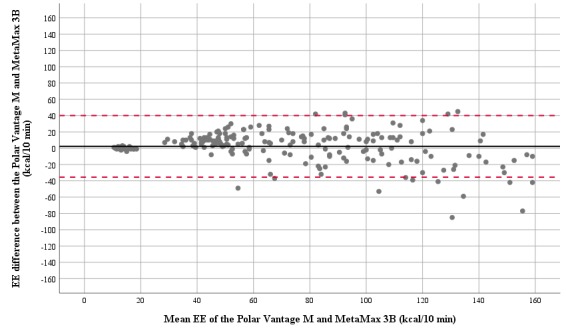
Bland and Altman plot of the energy expenditure (EE) estimation obtained during the 7 activity tasks (208 measurements). The solid line represents the systematic bias; the dashed lines represent the limits of agreement (systematic bias, SD 1.96).

**Figure 2:**
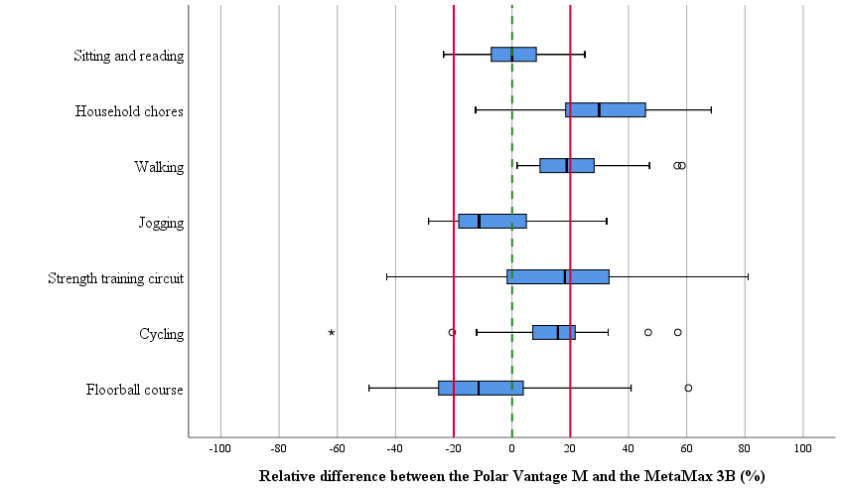
Relative deviation of the energy expenditure (EE) estimation by the Polar Vantage M compared with the criterion measurement of the MetaMax 3B separated for each activity task. The red lines indicate the proposed equivalence zone (SD 20% of the mean); the lower and upper boundary of the boxplots indicate the 25% and 75% quantiles of EE data, respectively, and the middle notch indicates the median data value. The whiskers include all data points that fall within the 1.5 interquartile range of the 25% and 75% quantile values. Circles and stars indicate EE data points that lie beyond the 1.5 and 3 interquartile ranges, respectively.

Each participant’s characteristics are presented in Table 1. The MetaMax 3B and Polar Vantage M reported the mean (SD) of EE to be 69.1 (42.7) kcal and 71.4 (37.8) kcal per 10-min activity, respectively (Table 2), with a correlation of *r*=0.892 (*P*<.001). Measured EE ranged from 10 to 194 kcal per 10 min, with the highest EE values obtained during the floorball course activity and the lowest EE values obtained during sitting and reading. The systematic bias was 2.3 kcal (3.3%), with 37.8 kcal limits of agreement (Figure 1). The mean absolute percentage error (MAPE) of the Polar Vantage M was 20.6%, ranging from 9.1% to 31.4%. On average, 59.5% of the mean EE values were accurate to within 20% when compared with those of the MetaMax 3B (Table 2). The household chores revealed the lowest accuracy (26.7%), whereas the sitting and reading revealed the highest accuracy (93.3%; Figure 2). Owing to its multicollinearity with perceived exertion (*r*=0.866; *P*<.001), the variable HR had to be excluded. A significant regression equation was revealed (*F*_2,207_=42.628; *P*<.001), with an R^2^ of 0.29. The perceived exertion (Borg scale value; odds ratio [OR] 2.028; *P*<.001) and wrist circumference (OR −1.533; *P*=.03) predicted 29% of the MAE within the Polar Vantage M.

Both predictors induced an underestimation of EE. The final linear regression models for clustered activity groups were similar to the data presented for the overall activities. The remaining independent variables, perceived exertion and wrist circumference, explained about 20% and 15% of the MAE in clustered low-to-moderate-intensity and high-intensity activities as well as 44% and 17% of the MAE in activities with no-to-little (steady) arm movement and a lot (unsteady) of arm movement.

**Table 1:** Participants’ characteristics.

Characteristics	Female (n=15), mean (SD)	Male (n=15), mean (SD)	Differences	*P* value	Overall (N=30), mean (SD)
Age (years)	30.1 (1.7)	28.9 (1.8)	−1.3	.51	29.5 (5.1)
Body height (m)	1.7 (0.1)	1.8 (0.1)	0.1	<.001	1.7 (0.8)
Body weight (kg)	60.2 (4.5)	74.8 (4.7)	14.7	<.001	67.5 (8.7)
Body mass index (kg/m^2^)	21.1 (1.3)	23.5 (1.5)	2.4	<.001	22.3 (1.8)
Wrist circumference (cm)	15.5 (0.8)	17.1 (0.9)	1.6	<.001	16.3 (1.2)
Skin hair on the wrist^a^	0.0 (0.0)	0.3 (0.5)	−0.3	<.001	0.2 (0.4)
Skin color^b^	2.9 (0.4)	2.9 (0.4)	0.0	>.99	2.9 (0.4)
Maximal oxygen uptake (mL/min·kg)	49.7 (6.0)	57.2 (5.5)	7.5	<.001	53.4 (6.8)
Resting heart rate (bpm^c^)	54 (10)	53 (7)	−0.7	.82	54 (9)
Maximal heart rate (bpm)	181 (11)	189 (9)	7.8	.04	185 (11)

^a^0=little hair, 1=moderate or a lot of hair.

^b^Fitzpatrick scale 1 to 6.

^c^bpm: beats per minute.

**Table 2:** Perceived exertion, heart rate, measured energy expenditure, and error rates of the Polar Vantage M when compared with the MetaMax 3B.

Activity	Borg scale (6-20), mean (SD)	% HR_max_^a^	EE^b^ by MetaMax 3B (kcal), mean (SD)	EE by Polar Vantage M (kcal), mean (SD)	Systematic bias in kcal, (limits of agreement)	Mean absolute error in kcal, (mean absolute percentage error)	20% accuracy^c^, n/N (%)
All activities	10.9 (3.5)	59.6	69.1 (42.7)	71.4 (37.8)	2.3 (37.8)	14.0 (20.6)	123/208 (59.1)
Sitting and reading	6.1 (0.3)	34.0	13.7 (2.5)	13.6 (2.1)	−0.1 (3.1)	1.2 (9.1)	28/30 (93)
Household chores	7.6 (1.0)	45.7	39.1 (8.6)	50.1 (10.6)	11.0 (16.5)	11.8 (31.4)	8/30 (27)
Walking (5.4 [0.5] km/h)	8.5 (0.9)	46.3	43.4 (6.8)	52.3 (8.5)	9.0 (12.3)	9.0 (21.4)	15/30 (50)
Jogging (9.6 [1.2] km/h)	11.8 (1.0)	71.4	113.7 (26.7)	103.3 (21.5)	−10.4 (33.5)	16.9 (14.8)	21/30 (70)
Strength training circuit (5.1 [2.8] kg)	13.5 (0.9)	63.3	61.8 (16.9)	71.0 (18.5)	9.2 (29.7)	14.3 (25.2)	15/30 (50)
Cycling (130.7 [22.6] W)	14.1 (1.1)	72.9	90.2 (15.5)	102.0 (24.9)	11.8 (34.9)	18.0 (20.6)	20/30 (66.7)
Floorball course	15.1 (1.7)	83.4	125.5 (36.1)	110.1 (29.3)	−15.4 (63.0)	27.5 (21.8)	16/28 (57.1)

^a^%HR_max_=percentage of maximal heart rate.

^b^EE: energy expenditure.

^c^Percentage at which the EE estimated by the Polar Vantage M was within 20% from the criterion MetaMax 3B.

## Discussion

### Principal Findings

In this study, a recently launched multisensory wristwatch, the Polar Vantage, was evaluated. The accuracy of the Polar Vantage was investigated in a simulated free-living environment at rest and during exercise by comparing the EE estimations of the watch with those of indirect calorimetry. The results revealed a systematic bias of 2 kcal per 10 min (−15 to 12 kcal/10 min) and an MAPE of 21% (9%-31%) during the different activities ranging from sitting and reading to a floorball course. Previous investigations demonstrated higher EE estimation errors of 14% to 210% during walking, running, and sitting [10]; 9% to 43% during lying, sitting, walking, running, and cycling [13]; and 9% to 24% during 13 activities ranging from those of low intensity (eg, writing at a computer) to those of vigorous intensity (eg, elliptical exercise or Wii tennis play) [27]. Shcherbina et al [12] investigated 7 wrist-worn monitors, and the MAPE values in EE estimation ranged from 27% to 93%, depending on the device. As such, these authors claimed that no wrist-worn monitoring devices in 2016 reported EE within an acceptable error range under controlled laboratory conditions during walking, running, and cycling.

Extending from these studies, this study assessed the performance of wrist-worn technologies estimating EE during a larger variety of low-to-high-intensity activities combined with little to a lot of nonsteady wrist and arm movement in a simulated free-living environment. Notably, the present findings are comparable with or better than those found in other recent studies on measurement systems that are also used as reference devices for EE measurement [11,28]. The wearable electrocardiogram Actiheart showed a similar MAPE in EE estimation of 20% (SD 15%), and the temperature- and acceleration-based SenseWear armband showed an MAPE of 39% (SD 18%) in semistructured activities [11,28,29]. To improve EE prediction, individual calibration is needed before each measurement with Actiheart, which hampers its feasibility of use in daily life. In contrast, the findings of this study represent a good overall EE accuracy in the Polar Vantage wristwatch, with the added advantage of its high ease of use.

In this study, larger wrist circumference and higher activity intensity (quantified as perceived exertion) were shown to predict an increased MAE in EE estimation. The highest Borg scale values were reported during the high-intensity activities, demonstrating diminished EE accuracy. This was in contrast to previous studies showing more accurate values during high-intensity activities than during low-to-moderate-intensity activities [10-13]. Noticeably, higher perceived exertion was reported in activities with mainly a lot of wrist and arm movement. Contrary to the fact that higher activity intensity predicted higher MAE, the Polar Vantage showed the very highest error rates during household chores, a low-to-moderate-intensity activity. However, household chores induce much arm movement. This was in line with the results of previous investigations, demonstrating that activities with more wrist and arm movement reveal increased error rates in HR estimation [12,14]. According to the manufacturer, Polar Electro Oy, EE estimation by the Polar Vantage wristwatch is based on the HR measurement and 3D accelerometer signal, with an activity-dependent weighting of these 2 components. During low-intensity activities, more acceleration information is taken into account, and during high-intensity activities, more HR information is taken into account. Moreover, they stated that challenges to accurate HR measurements include the hands facing down, wrist movement, cold skin, and incorrect device placement. Therefore, it is reasonable to state that the EE estimation of the Polar Vantage—and, most likely, many other wrist-worn monitors—is of reduced accuracy in activities that require strong nonsteady wrist and arm movement, regardless of the exercise intensity. Furthermore, EE is dependent on many anthropometric characteristics of the user [30]. In our study, larger wrist circumference revealed an increased MAE in EE estimation, which is in line with previous findings related to wrist-based HR assessments [12,14].

### Practical Implications

Generally, the Polar Vantage wristwatch showed promising accuracy in the estimation of EE. However, in activities with strong arm and wrist movements, the EE estimation remains challenging. In some activities, the arm with the mounted monitor takes an active part in the activity, whereas in other activities, it has a passive role. Second, in some activities, the human body is concentrated on doing physical work for a long time in a steady condition, which makes the physiology and calculation of EE more stable than it is in other activities that require a lot of stop and go or require little movement. On the basis of the present findings, the accuracy in EE estimation by the Polar Vantage is activity dependent, and we did not observe a tendency to either under- or overestimate EE. The Polar Vantage recorded EE during the sitting and reading activity—an activity task that is predominantly done during the day—with an acceptable accuracy in 93% of cases. However, the EE during household chores—a compulsory activity for many people and one that is often performed—was poorly assessed. On average, the wristwatch gives a valuable quantification of the training intensity and is a useful indicator of the daily energy output of a person. Nevertheless, such a monitor does not yet give accurate medical guidance or coaching on parameters such as how much one should eat for a balanced energy input and output.

### Limitations

The measurements were conducted after the specific exercise modes were selected for each investigated activity, which were ideal setups for testing the monitor. As such, the EE estimations presented in this study were obtained in a training mode and may look different from those measured during a 24×7 assessment of daily life.

### Conclusions

This study demonstrated that the multisensory wristwatch Polar Vantage has a statistically moderate-to-good accuracy in EE estimation that is activity dependent. During the sitting and reading activities, the EE estimation is good, whereas during nonsteady activities entailing wrist and arm movement, the EE accuracy is still moderate. However, compared with the other available wrist-worn EE monitors, the Polar Vantage can be recommended as it performs among the best. To better understand possible inaccurate measurements, users should be aware of the challenges that such technologies must still overcome.

## Abbreviations

EE: energy expenditure
HR: heart rate
HR_max_: maximal heart rate
MAE: mean absolute error
MAPE: mean absolute percentage error
OR: odds ratio
PAR-Q: physical activity readiness questionnaires
VCO_2_: carbon dioxide production
VO_2_: oxygen consumption
VO_2max_: maximal oxygen uptake
